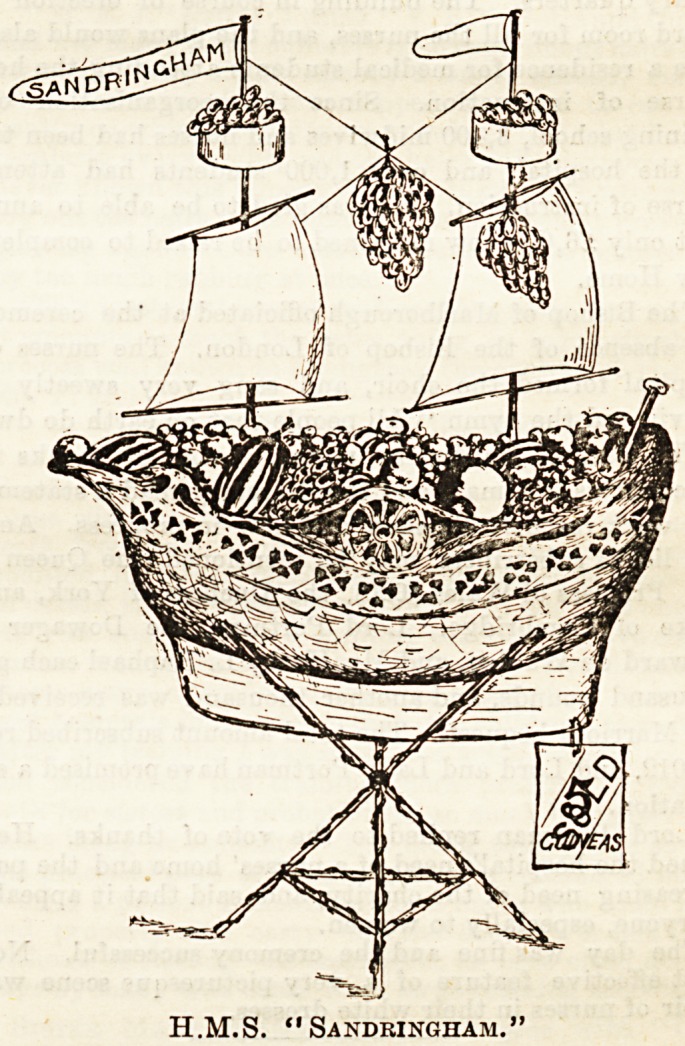# The Hospital. Nursing Mirror

**Published:** 1898-07-09

**Authors:** 


### "The Hospital" Nursing Mirror.

Being the Nursing Section of "The Hospital."

[Contributions for this Section of "The Hospital" should be addressed to the Editor, The Hospital, 28 & 29, Southampton Street, Strand, London, W.C., and should have the word "Nursing" plainly written in left-hand top corner of the envelope.]

### News from the Nursing World.

### HONOURS FOR INNISKEA NURSES.

The nurses who braved so much to carry succour to the typhus-stricken peasantry of Inniskea (of which our correspondent gave a vivid description in the "Mirror" for October 2nd, 1897) have been enrolled, by sanction of the Queen, Honorary Serving Sisters in the Grand Priory of the Order of the Hospital of St. John of Jerusalem in England. Their names are: Honoria Kenny, Frances D. M'Alister, Elizabeth Carson, Grace Simpson, Elizabeth Doyle, Flora Fitzmaurice, Margaret M'Mann, Mary Simpson, May Talbot, Sarah Caldwell, and Kate M. Kinsella. The sympathy and appreciation of Her Majesty and her family with those who face danger and hardship in the fulfilment of duty is well known; and all nurses will rejoice at the honours, worthily won, that have been bestowed upon their Irish sisters.

### LUCK FOR THE NURSES AT THE "LONDON."

The nurses at the London Hospital are indeed in luck's way of late. The newest addition to their comfort is one at which their sisters of a former generation would open their eyes in genuine astonishment. Several members of the committee have joined together and arranged for the complete redecoration and enlargement of the old sitting-room, now a really beautiful drawing-room, charmingly and cosily furnished, where tired workers may rest themselves in the depths of capacious couches and arm-chairs. The walls are wainscotted with white painted wood: there are bookcases for "light literature," writing-tables in the windows, a new piano, pretty rugs on the druggetted floor, and the aforesaid well-stuffed couches and chairs in most inviting profusion all about. A cunningly arranged mirror deludes the visitor into the belief that the room, large as it already is, is double its proper size, and the whole effect is delightful. Across the passage a second room has been fitted up as a library and reading-room, where nurses can secure quiet times for study. Another pleasant thing to record is the kind offer of Mr. Vacher, the vicar of St. Philip's Church, at the back of the hospital, to make over his beautiful garden to the free use of the nurses, only on the understanding that it is kept up by the hospital. The garden is only separated by the road from the hospital grounds, and as these latter will be spoilt for recreation purposes during the time that the alterations to the building are in progress, it may be imagined with what gratitude this generous proposal has been accepted. Only those who have lived in Whitechapel can quite realise the joy of a garden to sit in on summer days.

### THE NIGHTINGALE HOME.

On Thursday, June 30th, the Secretary to the "Nightingale Fund," Mrs. Henry Bonham-Carter, and the matron, Miss Gordon, were "at home" at the Nightingale Home, St. Thomas's Hospital, from half-past four to seven o'clock. Amongst the matrons present were Miss Monk, of King's College Hospital; Miss Pine, from Westminster; Miss Gordon, from Charing Cross; Miss Bland, from Poplar; Miss Herbert, Worcester General Infirmary; and many old students. The day was fine, and the home and hospital showed to great advantage. The "Bijou" Orchestra gave an excellent programme in the gardens. Tea and refreshments were served in the dining hall, and the corridor was converted into a charming lounge, with numerous easy chairs and seats. The decorations were good, and the elegant toilets of the visitors and the fresh dresses of the probationers and nurses produced a gay and varied effect. There are 36 probationers and nurses now in training at the school. Since its opening, in 1860, 864 nurses have obtained appointments from it. Ten probationers are now lodged in Block III., which is next the Nightingale Home. Accommodation is thus provided to enable each probationer, excepting in very few instances, to enjoy the privilege of a separate bed-room. The rooms themselves are of good size and very high. Increasing the number of probationers has relieved the strain of work in the wards, and has proved most satisfactory. The probationers have a large recreation-room in the basement, which is also used for classes, where they are at liberty to follow their own devices in a great measure. Bicycles are not yet "stabled" at the home, and those who ride store them elsewhere.

### DORSET COUNTY HOME FOR NURSES.

The terms offered by the Dorset County Home are high enough to secure efficient nurses. The salaries range up to £40 a year, with uniform, and as the matron is endeavouring to obtain a staff of fully-trained women, the credit of the newly-established society promises to be good. The fees charged to the patients are moderate, ranging from 10s. a week (which may be remitted in necessitous cases) to the poor up to £2 2s. for mental or infectious cases and operations. We hear that the home itself is pleasantly situated, and has ample accommodation for bicycles; that many gifts of furniture have been received, but that easy chairs, pictures, and books are still needed to complete the comfort and prettiness of the arrangements. A grant of £40 from the County Council is being spent in training two young widows in midwifery, and, as both possess some general training, they will prove useful in district and cottage work. Several handsome donations have been made, including one of £500 from the Earl of Ilchester, another of £100 from the Marquis of Salisbury, and another of £20 from the Prince of Wales. The list of annual subscribers is not yet complete, but it reaches upwards of £200. The outlook of the new association is therefore hopeful, and it deserves success. But the fact that it serves a generally scattered and not wealthy district makes it essential to provide against the disadvantages caused by the necessarily low fees and heavy working expenses. The committee will do well, therefore, to work up the subscription list carefully.

### REGULATIONS FOR PLAGUE NURSES IN MADRAS.

THE Government of Madras has ordered that the pay of plague nurses, whether European or Eurasian, shall be Rs. 100 a month, with a daily allowance for board of Rs. 1.8.0., free quarters, lights, travelling expenses for joining their first appointments and subsequent transfers, and carriage hire when accompanying search parties, provided in the last instance their pay is debited to the local funds. It is only just that expenses incurred in the performance of their duties should be defrayed by the authorities employing them. We still receive queries relating to the employment of nurses for the plague. The demand for them is fully supplied, and as the epidemic is happily decreasing, many now on service will no longer be needed. The pay is by no means extravagant, and unless there are private reasons for desiring work in India, nurses would do well to turn their attention elsewhere. If any are desirous of obtaining work in India the best way to get it is to put one's name down on the reserved list at the India Office, or to leave it with the Colonial Nursing Association, the Imperial Institute, or with the Up Country Nursing Association, of which the hon. secretary is Major-General J. Bonus, The Cedars, Strawberry Hill. There are other associations, which advertise in our columns from time to time.

### EALING HOUSE.

ON Tuesday, July 5th, the Committee of the Ealing House Girls' Home, St. Mary's Road, Ealing, received visitors on the occasion of the annual reunion of old girls, when Lady Margaret Villiers distributed the prizes. Tea was served at twenty minutes to four, and entertainments, music, and games were provided. This home is one of the "National Refuges," of which the training-ships *Arethusa* and *Chichester* are better known examples. Four hundred boys are trained on the ships; 300 on the farm school at Bisley; 120 at the Twickenham Home; and 200 girls are educated in housewifery arts in the Homes at Sudbury, near Harrow, and at Ealing.

### HUDDERSFIELD NURSING INSTITUTION.

THE Huddersfield Sick Poor Nurses' Institution is the outcome of the Diamond Jubilee memorial, and the way it has been arranged was fully explained at the first annual meeting held at the Town Hall on June 28th; £2,911 were collected for the fund, of which sum £2,497 were invested to yield £80 a year. The nurses, four in number, are lodged in the Home, Clare House, Clare Hill, and have been employed for seven or eight months. Ample testimony as to the value of their work has been received, and there is a substantial balance to the credit of the institution. Colonel Freeman, who moved the adoption of the report, pointed out that the expenditure would be £400 a year, and, as only £300 was received from assured sources, he trusted that the public would inform themselves of the aims of the institution and give it the needed help.

### HACKNEY NURSES' CERTIFICATES.

DR. CURNOW gave a satisfactory report of the nurses' examination at the Hackney Workhouse Infirmary. The candidates had been prepared by Dr. Gordon and the matron, and out of 21 presenting themselves six were awarded first-class certificates, three obtaining special recommendation; eight gained second class; and the remaining seven third-class certificates. It is worthy of note that those in the third class were said to be good practical nurses, failing, however, to attain a higher place through the difficulty they experienced in expressing themselves in writing.

### DRIFFIELD NURSING ASSOCIATION.

THE manner in which failure may be anticipated in the working of benefit nursing associations is exemplified by the sixth annual report of the Driffield Nursing Association. It was stated that one out of every eight subscribers needed the "benefit" for which the contribution was paid, necessitating the employment of extra nurses, the wages and expenses thus incurred involving a deficit of £12 in the year's accounts. Of course, unprofitable seasons occur in every enterprise, and in the case of the Driffield Association the debt is comparatively small with an expenditure of £376, but before any association can regard its pecuniary affairs as in a thoroughly satisfactory state, the income ought to exceed the outgoings, if only by a small sum, and a reserve fund should be maintained.

### WALSALL NURSES.

THE Victorian Nursing Institution at Walsall is beginning work quietly, but with due regard to its development. Number 40, Bradford Street is to be taken at a rental of £30 a year, furnished at a cost of £100, and two nurses are to be installed as soon as possible. The expenses for the first year, estimated at £210, will be discharged by the committee. In the meantime the subscription list is to be worked up to meet expenses. It seems only fitting that a town which raised a statue to Sister Dora should make pro-provision for its district nurses.

### SHORT ITEMS.

NOTTINGHAM Board of Guardians has appointed its first night superintendent of the probationer nursing staff. Nurse Stephenson has been chosen for this duty, at a salary of £35 a year.—Nursing Sister A. A. Murphy, of the Army Nursing Staff, has been stationed at the Royal Infirmary, Phœnix Park, Dublin. Nursing Sister A. R. Rose-Innes has been transferred thence to the Cambridge Hospital, Aldershot.—Nurse Beardeau has been appointed superintendent at Doncaster Workhouse, at a salary of £30.—The chairman of the Bridgenorth Board of Guardians presented Nurse Wilde, on behalf of the Nursing Association, with a silver medal, in recognition of her excellent services since she has been in its employ.—Nurse Boyd, who is leaving the service of the Eltham Board of Guardians, was presented with a marble clock by the chairman of the Board, on behalf of the officers of the house.—The district nurse appointed for one year's trial at Kirby Lonsdale has, during the seven months she has been at work, been so great a help and comfort to the sick that steps are being taken to secure subscriptions to enable the committee to retain her services. —Princess Christian's Nurses' Fund has received £41 11s. 6d. from the balance of the Windsor Diamond Jubilee Commemoration Committee. The Royal Infirmary has been awarded a similar amount. Each institution has benefited to the extent of £491 by the Jubilee Fund.—A District Nurse Sunday Fund is an established event in the annals of Barmouth charities. Special preachers pleaded the cause of the District Nurse Fund at St. John's, St. David's, and Llanaber, and the collections realised larger sums than on any previous occasion.—The Goole Board of Guardians have added ten years to the service of Nurse Webb for her superannuation pension. Nurse Webb contracted erysipelas in the performance of her duties, and was thereby incapacitated for further work. She will receive 8s. or 9s. weekly.

### Post-Graduate Clinics for Nurses.

By a Trained Nurse.

### ARTIFICIAL METHODS OF FEEDING.—V.

The first step in the nasal feeding of children or the mentally afflicted is to wrap the patient up in a blanket, securely pinned, so that he may not use his arms as weapons of defence against the due taking of food.

The patient lying on the back, an oiled, warmed, soft catheter—the size depends on the age of the patient, but there is no advantage in having it too small—connected by means of a piece of glass tubing to a glass funnel, is passed quickly backwards along the floor of the nares. The nourishment is poured into the funnel, and a good meal may thus easily be given every three or four hours without trouble or disturbance of the patient. In withdrawing the catheter it is necessary to tightly pinch this to prevent the escape of any fluid remaining in the tube which might set up a pharyngeal irritation.

In all methods of artificial feeding the catheters, tubes, and funnel need complete sterilisation, but more especially so in cases of diphtheria, tonsilitis, quinsy, cancer, &c. It may be well, too, to remind the nurse that the mouth of a patient, even when fed by nasal or stomach tube, requires constant attention. Some nurses seem to be under the impression that because little or no food is given by the mouth that the cleansing of this is of little importance. It is always a matter of nursing importance, especially as throat and mouth trouble so often is the cause which leads to artificial feeding. The mouth should be constantly swabbed by means of absorbent cotton sponges fastened on to new wooden meat skewers, and a borax or Listerine mouth wash must be regularly employed. It should be noted whether the tongue is coated, and if it should be a tongue scraper made from a ten-inch piece of whalebone doubled back to form a loop may serve to remove unhealthy tongue secretions. It by no means follows that a patient's digestion will not be affected by an unhygienic mouth, because the food he takes is not passing through that mouth. Fermentative and unwholesome conditions of mouth, tongue, and pharynx will very soon communicate themselves to the stomach and interfere with healthy digestion.

Cream, eggs, cocoa, and milk, beef essences, &c., may be used in the multiple variations these permit, and if the digestion show signs of not being equal to the amount and quality thus administered peptonisation will solve the difficulty.

A practical point with regard to nasal and stomach-tube feeding presents itself and should be remembered by the nurse. Bearing in mind that the action of the saliva is almost entirely missing in nasal and tube feeding, starchy and farinaceous foods should either be given in the form of a malted preparation or malt extract must be added in suitable quantities. A certain amount of saliva is secreted and swallowed when food is taken nasally and by tube, but the amount is not nearly sufficient to cope with starchy substances. Where the stomach shows signs of weakness not overcome by pre-digestion, or where nausea is present, nasal or tube-feeding may be alternated with nutrient enemata.

Forced feeding by means of a stomach tube is employed in a variety of cases, many of which are apt to be met with by the private nurse, whose skill in tube feeding will be much appreciated by every medical man who may find it necessary to prescribe this. In hysterical patients who obstinately refuse food, the stomach-tube is called into requisition, and two or three applications of this very unpleasant remedy often proves a "cure" to the refusal of nourishment. Some hysterical patients on the other hand take a delight in the fuss and trouble shown on their behalf. It satisfies a morbid love of attracting attention.

In Weir Mitchell cases tube feeding is often employed to supplement the food taken normally, for some such patients are really too exhausted to take the trouble to eat enough. In fact, an extreme indolence resulting from nerve exhaustion leads many patients to neglect their food. In some such cases, and in the aged, an infant's feeding bottle affords a ready means of nourishing. Food taken thus requires so little exertion on the part of a patient. Through loss of teeth some sick people suffer from a semi-starvation, and the feeding bottle is eminently suited to such cases, for the suction encourages the flow of saliva and the food is taken slowly, and consequently is more likely to prove digestible.

In those forms of melancholia where aversion amounting to loathing is produced by the sight of food, tube feeding must be employed, as also in the insane and those suffering from stricture of the œsophagus. It is resorted to in some phthisical patients, who from exhaustion or want of appetite are not taking enough food, and show signs of a rapid emaciation. Entire loss of appetite is not necessarily accompanied by a corresponding loss of digestive capacity. And marvellous improvement soon shows itself in phthisical and neurasthenic cases when forced feeding is employed. Tube feeding may be combined with the washing out of mucous and other stomach secretions, in which case it is found that the best time to give a full tubal meal is about one hour after lavage. If the patient shows a tendency to bite the tube, or a nurse's fingers, nasal feeding is to be preferred. A small cork placed between the teeth is a protection from involuntarily biting on the part of nervous patients. In the case of delirium tremens patients the feeding tube is passed through the aperture existent in the gag which is generally used for such cases when the stomach pump is employed.

In patients with good digestions, owing to the trouble involved in stomach tube feeding, two very large meals may be given in the twenty-four hours—in others, feeding will be necessary every four to five hours. And the tube can be passed infinitely more easily while the patient is in a sitting position with the back pillow-packed. There is a great disadvantage in using a tube of small calibre, since this may so easily pass into the larynx and the nourishment may thus find its way into the lung instead of into the stomach, an accident which may prove fatal. A large tube, on the other hand, if it slip into the larynx will cause such severe coughing that it will soon be expelled. If the oiling of the tube tends to nauseate, white of egg may be used instead, and a practical point to remember is that it is best to fill the tube before passing it—not only to keep air from the stomach—but from the fact that the fluid which will at once run down the throat causes a series of "swallows" which act favourably in getting the tube easily down the œsophagus.

Stiff hard tubes are very unsuitable for use, and must be repeatedly soaked in very hot water so as to soften them sufficiently to prove comfortable to a patient so fed. Even though a nurse may be skilful with the tube, the doctor will usually pass this the first two or three times so as to give the patient confidence. And he will generally require the nurse to pass the tube once in his presence to assure himself that she is deft and skilled. In very nervous cases the throat is often painted with cocaine to allay sensitiveness. The amount of food given by these artificial methods must depend on frequency of feeding and the condition of the patient. As much as ten to fifteen raw eggs in two quarts of milk are sometimes given in the twenty-four hours to very weak and melancholic patients. But the doctor will give full directions on this point, which the nurse will carry out intelligently, seeing to it that the food thus ordered is given in its most digestible form, and administered with the utmost comfort to the patient.

### Notes on Gynæcological Nursing.

By ARTHUR E. GILES, M.D., B.Sc., F.R.C.S., Assistant Surgeon Chelsea Hospital for Women.

### I.—ANTISEPSIS AND ASEPSIS.

THE most important thing for a gynæcological nurse to know is the subject of the principles and practice of asepsis and antisepsis, for without such knowledge the nurse, instead of being a help to the surgeon and a blessing to the patient, is likely to be a hindrance to the former and a source of danger to the latter; and so at the outset I must devote some remarks to this subject. Surgical practice within the last 50 years may be said to comprise three epochs—the era of sepsis, the era of antisepsis, and the era of asepsis.

The era of sepsis was that which prevailed before the introduction of Listerism. I do not mean that in those days every case operated on became septic; but this result occurred in a large proportion of cases. There were two important consequences—first, a very high mortality after all but the simplest operations; and, second, a tedious and painful convalescence in many cases. For wounds, instead of healing up at once—"by first intention," as it is called—became the seat of suppuration; indeed, so common was this that it was almost recognised as a necessary stage in healing. Pus was expected to form, and if it was not too offensive in its character, the grateful surgeon spoke of it as "laudable pus." When there was a free exit for the pus recovery ensued, though often slowly and with unnecessary drainage of the patient's strength. If the pus had not a free exit, absorption was apt to take place, blood-poisoning set in, and the patient not infrequently died of "septicæmia."

The era of antiseptics began with the recognition of the fact that blood-poisoning was an infectious disease due to germs. The brilliant scientific researches of Pasteur in France led the honoured Lister to apply the teachings of science to surgery, and he sought and found in carbolic acid a poison that would destroy the germs of sepsis. This era was, therefore, characterised by chemical disinfection applied to instruments, dressings, the operator's hands, the part operated upon, and even to the air itself in the form of the carbolic spray. The results in the direction of the diminution of mortality were perhaps the most striking and marvellous thing that has ever occurred in the history of surgery, whilst the application of the principle to midwifery practice resulted in the saving of tens of thousands of lying-in women from that awful scourge, puerperal fever. "Antiseptic" means "capable of destroying germs," and as many antiseptics are necessarily very irritating to living tissues, their use in the form of spray, lotions, and dressings, brought in contact with fresh wounds, often caused much disturbance. So surgeons have sought and found a more excellent way, inaugurating the era of asepsis. "Asepsis" means "an absence of germs," and the principle underlying the aseptic system is that all germs should be destroyed before the instruments, dressings, surgeon's hands, &c., are brought in contact with the wound. Disinfection by chemicals has largely been replaced by disinfection by means of heat, used in the form of hot air, steam, or boiling water; and for this purpose various sterilisers are used. Of course, heat cannot be applied to everything; it is not possible to boil the surgeon's hands or the patient's skin, so that for many purposes chemical disinfectants are still required.

Now the main idea that I wish to impress upon every nurse who reads these notes is that germs, which are exceedingly minute living organisms, are found everywhere. They abound in the air and in dust of all kinds; they are found in quantities on and in the healthy skin, and even in the internal passages, such as the mouth, nose, and alimentary canal, and in the vagina in women. All germs are not equally dangerous. Some are quite harmless; but it is not possible to tell beforehand, without very special scientific methods, whether the germs in any given situations will be dangerous or not. So that it is essential that before an operation is begun, while it is being carried on, and after it is finished, there should be no possibility of any living germs being brought in contact with the operation wound. This is a very difficult thing to secure; and hence the minute and multitudinous precautions that have to be adopted.

Let me next impress the fact that ordinary cleanliness, although it is all-important in surgery, is not sufficient for the removal or destruction of germs. Hence everything that is likely to be brought into contact with an operation wound must be specially prepared. All instruments and dressings that will stand heat must be boiled or placed in hot-air stoves, and that not only for a minute or two, but as a rule for half an hour or an hour. All other things must be treated by chemical antiseptics. I need not here describe in detail the various methods and antiseptics employed; I rather wish to make quite clear what are the principles to be adopted. The details can be learnt from one of the many books on the subject. But one thing I must dwell on, and that is the question of the nurse's hands. The hands should be scrubbed with soap and hot water for five to ten minutes before touching either the operation-site on the patient or anything that is going to be used for the operation. The nails must always be kept short and must also be carefully scrubbed and cleansed. After the washing the hands must not be simply dipped, but must be soaked and scrubbed in some antiseptic such as corrosive sublimate 1 in 1,000, carbolic acid 1 in 40, or permanganate of potash. If the latter be employed mere Condy's Fluid is no use; the solution must be saturated, so that it stains the hands a deep mahogany colour; and the colour is then got rid of and the disinfection completed by dipping the hands in a saturated solution of oxalic acid. The disinfection should extend, not to the hands alone, but to the arms from above the elbows downwards. No rings should be worn, and the sleeves should be quite short. Clean linen over-all blouses or aprons should be worn over the ordinary clothes. For a nurse to assist at an operation with long sleeves and cuffs is quite against the principles of asepsis. After the hands have been washed, nothing should be touched that has not been disinfected; and so no ordinary piece of furniture, cans, bowls, or jars should be touched, nor should the handkerchief be used. A nurse with a cold in the head should not assist at an operation. If anything "septic," that is, which has not been specially disinfected, has to be touched, including the hands or clothes of the patient, the nurse should disinfect her hands again before touching any instruments, dressings, or sponges. Anything that drops on the floor must be left to lie there, or picked up by some person not assisting at the operation, and disinfected again before it is used.

Let the nurse remember that upon the conscientious attention to all these details the health, and, indeed, the very life of the patient may depend.

### CHARTS FOR NURSES.

WE are pleased to see a new edition of Miss C. M. Löhr's "Nurses' Report Book." Copies may be obtained from the proprietress, the Cottage Hospital, Potter's Bar, Middlesex, at 7d. a-piece, or 6s. 6d. a dozen. It is arranged for day and night reports for three weeks, and each copy contains a temperature chart.

### Nursing in India.

### NOTES ON CHOLERA AND SMALL-POX CASES.

(By an Indian Correspondent.)

Above all I dread those cases of cholera which linger on for some seven days, after which they die of blood poisoning. The high fever, delirium, and restlessness makes them such painful cases. During the cholera season last year we had several of this nature, and one dreads the gradual quieting down, which foretells the end.

One case, a fatal one, was that of a young woman about 23 years of age. She was pregnant; abortion, of course, occurred, and the placenta was adherent. Occasionally, when the patient is convalescing, hiccough is troublesome and persistent. Morphia, in such a case, is injected into the abdomen as a last resource, to prevent the patient dying from exhaustion. Two injections usually prove sufficient. The first case which came to us last year was sent in as a plague case. I very soon sent for the house surgeon, and the poor lad was removed to the proper wards, where he died that night. The next cholera case was not admitted until nearly a month later. This admission caused some consternation, for there were three plague patients in the same ward. This only continued for a short time, for our accommodation is limited, and as the number of plague patients increased they were taken straight to the regular hospitals.

We had quite an outbreak of small-pox in the beginning of the spring of this year. Amongst the cases were two Europeans, these being no less than a sister and a probationer belonging to our own hospital. The others, about 30 in number, were natives, and all made very good recoveries.

One, a woman, about 32 years old, was a very severe case. Her first known temperature was 100·6 deg.; there was then no rash, but a slight sore throat, and the characteristic backache. On the second day after admission temperature was 105·4 deg., and there was a decided rash all over the body; troublesome vomiting had been present all day, and the throat and backache became much aggravated. The patient was then removed from the observation to the small-pox wards. By the third evening the temperature had risen to 106·4 deg. She suffered violent delirium all day, vomiting continuing. Borax and glycerine was applied to the throat, and the eyes bathed frequently with boracic lotion. The eruption was now very marked, and it proved to be a confluent case; the patient was very restless. For the two succeeding days and nights the temperature ranged from 102·8 deg. to 105·6 deg., all the other symptoms continuing, and the rash was now fully out; vomiting ceased. For the next week temperature ranged from 99·8 deg. to 102 deg.

As usual in such cases, constipation proved very troublesome, necessitating doses of mag. sulph. every third or fourth day. The rash was now more or less "pustular," and many of the pustules were umbilicated. By the end of the third week the temperature remained below 99·4 deg., and gradually fell to much below normal; scabbing also began. Feeding proved troublesome, as the patient had no appetite; under a tonic, however, her strength gradually returned. She was discharged in the beginning of the eighth week.

Both the windows and glass panes of the doors of these wards are painted green; each ward has three or four doors, two big windows, and four ventilators. The rooms are very high, and unless obliged by pressure on beds, we do not put more than one patient in each. The floors are of asphalt, so that they can be washed down every morning with lotion. A calamine and eucalyptus lotion was painted on every day, and as often as it cracked, which of course it frequently did, and when scabbing took place, "cakes" of it fell at a time. Washed face for the first time in the sixth week. The body was gently sponged with eucalyptus lotion, which certainly kept the patient sweet and clean, besides allaying the irritation.

The first disinfectant bath was a great business, and it was a great relief to patient and nurse when it was over.

Natives do not regard small-pox as repulsive, and do not as a rule isolate such cases. They think they may as well have it and be done with it. In the large towns they are not afraid of vaccination, but in the villages the women take the children into the jungle when the district vaccinator goes his round.

### The Press Bazaar.

### THE PRINCE OF WALES'S GIFT.

We thought it would interest our readers to learn a little more as to the splendid gift of fruit made by the Prince of Wales to The Hospital stall at the Press Bazaar, and have

H.M.S. "Sandringham."

consequently had a sketch made of the "ship basket" referred to in our notice of the Bazaar last week. Our illustration will give a rough idea of this beautiful trophy, standing over 3 feet high, which contained some of the Sandringham fruit. From the mainmast waved the flag in scarlet and white. The fighting tops were filled with strawberries, and between the masts hung magnificent bunches of grapes. Peaches, nectarines, melons, and pineapples filled the ship in every corner, rendering its burden as costly as any galleon which ever sailed from the ports of Spain. It is not surprising that the Sandringham ship was one of the first purchases made at The Hospital stall. More superb fruit arrived from Sandringham on the second day and received artistic treatment at the hands of those entrusted with the decoration of the stall, but all agreed that the ship which had sailed to port was one of the most charming features of the Press Bazaar.

### Death in Our Ranks.

We regret to announce the death, on June 28th, of Annie N. S. Murray, aged 34, night superintendent of the Poplar and Stepney Sick Asylum, Bromley-by-Bow, E., late of the Birmingham General Hospital and Army Nursing Service.

### Queen Charlotte's Hospital.

### LAYING THE FOUNDATION-STONE OF THE NURSES' HOME.

THE foundation-stone of the new Nurses' Home for Queen Charlotte's Hospital was laid on Wednesday afternoon by Viscountess Portman. Lady Portman, who was accompanied by Viscount Portman, was received by the chairman, the Earl of Hardwicke, members of the committee of management and of the medical staff, and was presented with a bouquet of crimson roses by Miss Naomi Davies. The Earl of Hardwicke read a short address of welcome, in which he reminded Lord and Lady Portman that the late Lord Portman had also been president of the hospital. He said that the increased demands for accommodation for patients had necessitated nearly the whole of the nursing staff being lodged in temporary quarters. The building in course of erection would afford room for all the nurses, and the plans would also provide a residence for medical students attending the hospital course of instruction. Since the reorganisation of the training school, 3,400 midwives and nurses had been trained in the hospital, and over 1,000 students had attended a course of instruction. He was glad to be able to announce that only £6,000 now remained to be raised to complete the new Home.

The Bishop of Marlborough officiated at the ceremony in the absence of the Bishop of London. The nurses of the hospital formed the choir, and sang very sweetly Psalm cxxvii. and the hymn "All people that on earth do dwell."

The Earl of Hardwicke moved a vote of thanks to the Viscountess Portman, and the Secretary read a statement of the work recently finished and now in progress. Amongst the list of subscribers may be mentioned the Queen (£50), the Princess of Wales (£20), the Duchess of York, and the Duke of Cambridge. Lord Portman, the Dowager Lady Howard de Walden, and Mr. Henry L. Raphael each gave a thousand pounds, and another thousand was received from the Marriott bequest. The total amount subscribed reaches £8,012, and Lord and Lady Portman have promised a second donation.

Lord Portman replied to the vote of thanks. He mentioned the hospital's need of a nurses' home and the public's increasing need of the charity, and said that it appealed to everyone, especially to women.

The day was fine and the cremony successful. Not the least effective feature of a very picturesque scene was the choir of nurses in their white dresses.

### Where to Go.

### ROYAL BRITISH NURSES' ASSOCIATION.

WE are requested to announce that the annual meeting of the Royal British Nurses' Association will take place in the East Conference Hall of the Imperial Institute, on Tuesday, July 12th, at four p.m. Members are invited to a reunion afterwards in the Earl's Court Exhibition, where a special tea will be provided for members and their friends in the tea house in Picturesque England from half-past six. Tickets and all further particulars on application to the secretary, 17, Old Cavendish Street, W.

ROMFORD UNION WORKHOUSE.—Miss Ellen E. Wilson has been appointed Superintendent Nurse at this workhouse. She was trained and afterwards staff nurse at St. Marylebone Infirmary. She has been engaged as district nurse, Queen's nurse, and in private work.

### QUEEN VICTORIA'S JUBILEE INSTITUTE FOR NURSES.

At the request of the secretary of Q.V.J.I.N., we have much pleasure in stating that the name of Miss Elizabeth Haughton was unfortunately omitted from the list of nurses appointed "Queen's" nurses on July 1st. Miss Haughton is working at Bolton.

### Appointments.

### MATRONS.

A CORRECTION.—Miss Alice M. Raynes, whose appointment as Matron of the Cuddington Isolation Hospital, Surrey, we noted last week, informs us that she was "sister" at St. George's Hospital, Hyde Park Corner, S.W., after having been staff nurse; that she received her fever training at the South-Western Fever Hospital, Stockwell, and not Fulham, and that she has held her present position as assistant matron at St. George's Hospital for the last sixteen months. We are glad to have the fuller and more correct particulars.

LONDON ORPHAN ASYLUM, WATFORD.—Nurse Adela d'Arcy was appointed Nurse-Matron of this asylum on June 29th, 1898. She was trained at Sussex County Hospital, and her previous appointments have been as follows: Head nurse Suffolk General Hospital, night superintendent Worcester General Hospital, ward sister Aberdeen Royal Infirmary, night superintendent Wolverhampton and Staffordshire General Hospital, matron Andover Cottage Hospital, and nurse-matron London Throat Hospital.

SWANSEA GENERAL AND EYE HOSPITAL.—On July 1st Miss C. Rigney was appointed Matron of this hospital. She was trained, and afterwards in succession ward nurse, night superintendent, and assistant lady superintendent at the Royal Infirmary, Liverpool, during a period of twelve years. She was matron of the Victoria Hospital, Burnley, for several years.

QUEEN VICTORIA'S NURSING INSTITUTION, WALSALL.—Miss Eyre has been appointed Matron of this institution. She was trained at the Royal Manchester, and for district nursing at the Ardwick and Ancoates District Nurses' Home, Manchester. She has been on the staff of the Queen Victoria Nursing Institution, Wolverhampton, for three and a half years.

METROPOLITAN ASYLUMS BOARD.—Miss Ethel Julia Marion Keene was appointed Superintendent-Nurse of the Darenth Adult Asylum on July 5th, 1898. She was trained at the Children's Hospital, Shadwell, and at St. Bartholomew's Hospital.

PRINCESS ALICE MEMORIAL HOSPITAL, EASTBOURNE.—Miss Ramsay, who has been staff nurse of the above hospital for nearly five years, became Matron on June 24th in place of Miss Lambert, who recently resigned.

### Minor Appointments.

ST. LEONARD'S INFIRMARY, SHOREDITCH.—The following Charge Nurses have been appointed to this infirmary: Miss Ellen Naomi Nash, who was trained at the Central Sick Asylum's District Infirmary, Cleveland Street, and who has held subsequent posts at Poplar and Stepney and Bethnal Green Infirmaries; and Miss Julia Robinson, who was trained at the Royal South Hants Infirmary, and who was afterwards engaged in private work.

UNION INFIRMARY, SUNDERLAND.—Nurse Mary White has been appointed Charge Nurse at the above infirmary. She received three years' training at the New Infirmary, Birmingham, and for the last twelve months she has been engaged in private work at Newcastle-on-Tyne.

HACKNEY UNION INFIRMARY, HOMERTON.—Miss Glanville has been appointed Assistant Matron of this infirmary. She was trained at St. Bartholomew's Hospital, Cornwall, and has held posts in the Infirmary, Truro, and in the Convalescent Home, Parkwood, Swanley, Kent.

HASLINGDEN UNION WORKHOUSE INFIRMARY. — Miss Catherine Cormode was appointed Superintendent Nurse of this infirmary on June 29th, 1898. She was trained and afterwards charge nurse at Salford Union Infirmary.

### "The Hospital" Convalescent Fund.

WE have just received a welcome contribution from Miss Briggs to THE HOSPITAL Convalescent Fund of 4s. We should be glad of further contributions as the balance in hand is by no means large, and we should not like to be obliged to refuse help to any deserving case. The summer weather is now overdue, and naturally everyone is thinking of change and rest, whilst those who have been ill certainly require it.

### Japan.

### THE INTRODUCTION OF TRAINED NURSING.

THE marvellous adaptability displayed by the Japanese in adopting Western civilisation renders the following article relating to the introduction of nursing, which appeared in the *Weekly Sun* of the 26th, interesting in an unusual degree:—

"As a rule," says Miss Fraser, "the Japanese women make very good nurses. They are light and quick of foot and very deft with their fingers. Before a nursing school was opened, twelve years ago, there were no trained nurses in Japan. There were old women, who took care of sick people, and there were men nurses for the male patients in the hospitals.

"At first it was not easy to get a good class of women. The Japanese looked upon nursing as a menial form of labour, and the educated women felt that they were above such a calling. In time, however, they found out that nursing was a profession requiring skill and intelligence, and that it was regarded by foreigners as distinctly creditable. They form their ideas on those of foreigners nowadays, so it wasn't long before we were able to get our students from a higher grade of women. They receive what seems to be slight compensation; but over there, where living is so cheap, it is very good pay. They have a little less than two shillings a day for any time under two weeks. There is a slight reduction for a longer time.

"When we first went to Japan, of course their methods were totally unlike ours; but many of their physicians have studied of recent years in England, Germany, and America, so they have made great progress. The most common ailments are skin diseases and consumption. They have a good many eye troubles, too. Their food is not nourishing, and has not been so for generations, so that their constitutions are not strong. They do not have enough warmth in winter. It is really cold, and their houses, which are mere shells, are scarcely warmed by the little bit of charcoal in the middle of the room.

"We took stoves with us and a great many other foreign comforts and conveniences. But you have no idea how difficult it was to teach the Japanese nurses how to care for the sick. I had to begin my teaching from a point entirely different from the one to which I was accustomed; the houses, the heating arrangements, the clothing, the beds, the diet are different. In the hospital we arranged most of these things to suit ourselves, but in private nursing it really was like entering a strange country.

"Even in the hospital we had hard work to arrange a diet for the sick. The people there eat almost nothing but rice and fish. I had all my recipes with me, but I had to devise an entirely new set. In case of typhoid fever, for instance, of course we tried to prevail on them to take a milk diet. But they don't like milk. They have always had a superstition against eating anything which has had animal life. They include milk and eggs in this category.

"Some of them will consent to take milk now, but when they absolutely refused I made them a broth of rice, boiling it until it was soft, and then draining the liquor off. I could get chickens, and so we could make chicken broth in cases where it was needed. There was no lamb or mutton, there being no sheep in Japan. We could get beef in the cities where there was a foreign population. The Japanese care little for fruit. They like the little mandarin oranges, and that was a good thing, for we had them about eight months out of the year. They have persimmons, too, and figs; but they don't care much for the figs; they say they are 'children's fruit.' The plums there do not amount to much. They have a sort of pear which looks like an apple and tastes like wood. As for the famous cherry trees, they are cultivated for their blossoms, not for their fruit.

"It is only in the cities that the people are beginning to use knives and forks and spoons. As a general thing, even among the more progressive Japanese, they are not used, while in the country you see nothing but chopsticks and drinking dishes. It is not so difficult for them to eat rice with chopsticks as one would imagine. They do not cook it as we do. They cook it just enough to make it sticky, and then it isn't hard to pick up a lump with their chopsticks. They do not put any salt in it, either, so you can imagine how palatable it is.

"The uneducated people are full of superstition, especially about sickness and death. If they have, for example, anything the matter with the ear, they make a wax model of an ear, put it up in the temple, and pray to it. They can construct a god to fit any complaint, you see. They have learned a great deal, however, in the last ten years."

### Everybody's Opinion.

### HOLIDAY MAKING.

NOVELTY HUNTER writes: I am wishful to have a new kind of holiday. I have a very light district in lovely country, and a month's holiday due any time now. Do you think it would be a safe venture to try and get a berth as nurse on board, to go abroad anywhere? Should I receive any pay, and do you think it would be nice. Will you please tell me how to set about getting information.

[Our best reply to "Novelty Hunter" is to put her letter into "Everybody's Opinion," and invite other nurses in similar circumstances to give their experience.—ED. *T. H.*]

### SUPERFLUOUS HAIR.

"M. E. J." writes: I think "V. K." (Notes and Queries 118) and her many sister sufferers from superfluous hairs cannot be aware of a very simple remedy I learnt abroad, viz., with a piece of fine pumice stone dipped in water to rub the skin gently on which the hairs are growing; this must be repeated regularly every night, and the hairs will disappear. I have never known this remedy to fail. It may be hastened by pulling out the hairs; the pumice stone treatment prevents their growing again. Care must be taken not to redden the skin by too much rubbing at once.

[This treatment cannot eradicate the roots, and we fear that it amounts to much the same thing as regularly shaving, a process well known to strengthen the growth of the hairs.—ED. *T. H.*]

### MATRONS' COUNCIL MEETING.

"L. S." writes: I am greatly interested in "A Constant Reader's" opinion of training in hospitals of 40 beds, and agree with her that in many instances very good nurses go forth from such, and it is hard that the time spent is not counted in larger institutions. The work is more practical in detail, the probationers having "to do," not only "look on," as one does in a medical school. I have seen many nurses trained in small hospitals that would have put to shame many a gold medallist. I was trained in two of the best hospitals in London: first for children, second adults. After my training I was sister in a small hospital of 80 beds, and considered the training more practical in every way, both for sisters and probationers, so much being left to them, there being no students. Too much theory and so little of the practical part of a nurse's duty is considered nowadays. Were I a patient I should prefer a nurse who could make my bed properly and carry out the doctor's orders faithfully without criticising the treatment, as is so often done nowadays by many who come from large training schools.

SISTER MARY writes: I quite agree with "Constant Reader" in last week's HOSPITAL as to a more practical training being got in a hospital where there are no students, for the nurses have the dressings to do. Where I was trained there were about eighty beds, and only one house surgeon; the staff nurses of each ward had to prepare the theatre for their own patients; the senior night nurse had to attend to the accidents and to prepare the theatre when required during the night. We had lectures given by the house surgeon and the matron; we had also practical lectures given in the wards by the matron. As for the London-trained nurses, I admit they may know more theory, but the country nurse gets more practical work. It is not passing examinations—no, not even with honours—that makes a good nurse, for unless she be observant, conscientious, self-denying, and patient, all the lectures she may attend will not fit her for the noble work she has undertaken to do. I think, if some of the London matrons were just to go through some of the country hospitals, they would find that the country matrons' training were quite equal to their's—not better, naturally. I wish some other nurses who have had the pleasure (not misfortune) of being trained in a country hospital would give a little experience of their training, and, perhaps, at the next Matrons' Council meeting the matrons may do more justice to their country sisters.

### For Reading to the Sick.

"THERE SHALL BE NO MORE TEARS."

**Verses.**

CAME morning, can I tell,
How this poor frame it's sorrowful tenant kept?
For waking nights were mine, I sleeping wept,
And days, months, years, that sorrowful vigil kept;
Alas! Farewell.
How often it is said,
I sit and think, and wonder too sometimes,
How it will seem, when in that happier clime
It never will ring out like funeral chime
Over the dead.
No tears! no tears!
Will ever a day come that I shall not weep?
For I bedew my pillow in my sleep;
Yes, yes, thank God, no grief that clime shall keep,
No weary years.
Aye, it is well!
Well with my lambs, and with their earthly guide,
There, pleasant rivers wander they beside,
Or strike sweet harps upon its silver tide.
Aye, it is well.

Pray! though the gift you ask for
May never comfort your fears,
May never repay your pleading—
Yet pray, and with hopeful tears!
An answer—not that you long for,
But diviner—will come some day;
Your eyes are too dim to see it,
Yet strive and wait and pray!
—*A. Procter.*

Father of all, to Thee
We breathe unuttered fears.
Deep hidden in our souls,
That have no voice but tears;
Take Thou our hand and through the wild
Lead gently on each trustful child. —*Julian.*

I thank Thee more that all our joy
Is touched with pain;
That shadows fall on brightest hours,
That thorns remain;
So that earth's bliss may be our guide,
And not our chain.

**Reading.**

"These are they which came out of great tribulation—and God shall wipe away all tears from their eyes."

An old promise, given long ago, and renewed in the last chapter of the Bible. God is waiting patiently for that day, and He expects us to wait patiently, too. Though His heart of Fatherly love is longing to set us free, He will not act too soon. Each burning drop that falls from the aching heart has its work to do. But soon the time shall come, and then "There shall be no more sorrow, there shall be no more crying." O blessed words! Who can measure their worth? Is there ever a moment on earth when God, looking from His throne above, does not see the glitter of countless tear-drops, falling here, falling there, shed easily by the little child; shed with struggling sobs by the sorrowful woman, shed in reluctant anguish by the strong man? No, not countless, for God counts them all, and no earthly heart, though of the tenderest, can see those tears with the measureless pity which fills our Father's heart as He gazes—yet He lets them fall, He works no miracle to remove the cause. The strong, toothed wheels of life grind onward, and tear after tear must sink into the dust.

For a little while. Not for ever. Soon, very soon, "God shall wipe away all tears."—*Agnes Giberne.*

### Notes and Queries.

*Blackheads.*

(136) How can I get rid of blackheads? I have tried hot water and sponging, scrubbing, squeezing, lotions, &c., but have found them useless.—*An Anxious Nurse.*

We refer "An Anxious Nurse" to our rules relating to correspondence at the head of this column.

*An Appointment as Nurse-Matron.*

(137) Kindly tell me how I may obtain an appointment as nurse-matron? I hold a two-year certificate from a large provincial hospital, I have excellent testimonials from the chairman and officers of the infirmary where I held this position for two years and six months. I have answered advertisements and advertised myself, but to no purpose. —*A Perplexed One.*

Your difficulty is evidently that you have not a three-year certificate. The recent Local Government Board Order makes this qualification essential for a superintendent nurse or matron. Did you leave your training school at the expiration of the two years' training? If you spent a third year in its service the authorities might possibly give you a certificate for three years. If not, could you not return for another year's service, arranging that a three-year certificate should be given you at the end of the time? Your case is undoubtedly a hard one, and if you will send us the dates of your training and subsequent appointments and copies of your testimonials we will make inquiries as to what you ought to do.

*Linen Maid.*

(138) Would it be possible to obtain a position as linen maid in a hospital without previous experience? Could you give me a hint as to what the duties are? Might I advertise in the "Mirror" for such a post.—*Ignoramus.*

You might advertise for such a post, but we doubt if anyone would care to employ an untrained person. We should advise you to apply for the post of assistant matron in a boys' school, taking a small salary until fully competent. You must, of course, be a good needlewoman, able to mend, mark, and fold linen properly.

*Plague Nursing.*

(139) Kindly give me some information on plague nursing? Who engages the nurses, and for how long? What qualifications must they have?—*A. M.*

The Revenue Secretary, the India Office, engages the nurses sent out from England to nurse the plague. Full particulars of terms of engagement are printed in "The Mirror" for October 23rd, 1897, p. 38.

*The Value of Certificates.*

(140) Can a committee or doctors of a cottage hospital grant a certificate to probationers or nurses, such as would be of service to them, where the doctors as well as the matron undertake weekly lectures? If so, after what length of training?—*Inquirer.*

For the post of superintendent nurse under the Poor Law, and for membership to many important associations, a certificate of three years' training from a hospital recognised as a training school possessing a resident medical officer is essential. The value of the certificate, therefore, depends very much upon the work you intend taking up. We always advise the recognised training.

*Free Convalescent Home.*

(141) Could you tell me if there are any free convalescent homes for nurses, or of any place where an invalid nurse could stay for a time at a very moderate charge?—*K. E. T.*

If rest and change will enable "K. E. T." to resume nursing work, and her case can be properly recommended, she is eligible for "THE HOSPITAL Convalescent Fund." She must then send a doctor's certificate and two references to householders to the secretary. The Merchant Taylors' Convalescent Home (particulars from Edward Nash, Esq., 30, Threadneedle Street, E.C.) and the Thomas Banting's Memorial Home, Worthing (apply the secretary), are both free.

*Partially Trained.*

(142) Is there a hospital or nurses' home at Tynsdly, Lancashire? Please give the name of a hospital which will receive a partially trained nurse to work for a three-year certificate?—*Olga.*

We do not know of a hospital at Tynsdly. (2) It will be difficult for you to enter a hospital, as many matrons object to the partially trained. The Matron, Victoria Park Hospital, might be able to. See our advertisement columns, and if there is nothing in them suitable advertise yourself.

*Fresh Air Cure.*

(143) Are there any sanatoriums in England conducted on the same system as the German "Fresh Air Cure" treatment for consumption? (2) Are there any establishments for the cure of consumptives in England, and where are they situated?—*P. B. (Ratonagh).*

Only a few private establishments have as yet begun this treatment, though at the National Hospital for Consumptives, Ventnor, the treatment includes it to a great extent. (2) See Burdett's "Hospitals and Charities" for list of institutions for consumptives.

*Age.*

(144) Is the candidate of the Medico-Psycological Examination asked to give her age?—*Inquirer.*

Candidates are not required to give their ages, but it is not usual to accept any under that of 21 years.

*Children needing Massage.*

(145) Can you tell me of an institution where children needing massage and requiring to lie down a great deal can be received?—*Auntie (London).*

See Burdett's "Hospitals and Charities." You may consult a copy at this office if you wish.

## Figures and Tables

**Figure f1:**